# Parameters Affecting Nausea and Vomiting After Thoracoscopic Wedge Resection in Patients With Pneumothorax

**DOI:** 10.7759/cureus.19926

**Published:** 2021-11-26

**Authors:** Musa Zengin, Hilal Sazak, Ramazan Baldemir, Gulay Ulger, Semih Aydemir, Leyla N Acar, Ali Alagoz

**Affiliations:** 1 Anesthesiology and Reanimation, University of Health Sciences, Ankara Atatürk Chest Diseases and Thoracic Surgery Training and Research Hospital, Ankara, TUR; 2 Thoracic Surgery, University of Health Sciences, Ankara Atatürk Chest Diseases and Thoracic Surgery Training and Research Hospital, Ankara, TUR

**Keywords:** pneumothorax, visual analog scale, platelet/lymphocite ratio, neutrophil/lymphocite ratio, postoperative nausea vomiting

## Abstract

Background: Postoperative nausea and vomiting (PONV) is one of the complications that can occur frequently in the first 24 hours postoperatively. We aimed to investigate the parameters that could predict PONV in patients who underwent thoracoscopic wedge resection for pneumothorax.

Materials and Methods: After obtaining the approval of the ethics committee (ID: 2012-KEAK-15/2358, Date: 14.09.2021), the records of patients who underwent elective video-assisted thoracic surgery (VATS) between January 2018 and June 2021 were analyzed retrospectively. The patients who underwent elective thoracoscopic wedge resection for pneumothorax, who were between the ages of 18-65, American Society of Anesthesiologists (ASA) I-III, and whose body mass index (BMI) was between 18-30 kg/m^2 ^were included in the study. However, patients who received a blood transfusion or used antiemetics, anticholinergic drugs, and analgesics continuously were not included. In addition, patients with a history of chronic pain were not included in the study. The patients were divided into two groups, the PONV group (Group 1) and the control group (Group 2). The PONV incidence, visual analog scale (VAS) scores, 24-hour morphine consumption, additional analgesic requirement, neutrophil/lymphocyte ratios (NLR), and platelet/lymphocyte ratios (PLR) were evaluated.

Results: The groups were similar in terms of demographic data (p > 0.05). Additional analgesic requirement and 24-hour morphine consumption were significantly higher in the PONV group (p: 0.005, p < 0.001, respectively). Preoperative NLR (p < 0.001), postoperative NLR (p < 0.001), preoperative PLR (p < 0.022), the VAS scores of the first hour (p: 0.004), and 24^th^ hour (p < 0.001) were statistically significantly higher in the PONV group compared to the control group.

Conclusions: NLR parameters can be effective with high sensitivity and specificity in predicting PONV during the preoperative and postoperative period. Besides, preoperative PLR may also be effective in predicting PONV. A treatment that can be planned according to these parameters may play a key role in preventing PONV. In addition, efficient perioperative analgesia management may be effective in reducing PONV by limiting the emetogenic analgesics.

## Introduction

Postoperative nausea and vomiting (PONV) is one of the most common complications defined as nausea and vomiting that develops within the postoperative 24 hours [1,2]. PONV is associated with negative effects ranging from pain at the suture line to postoperative morbidity, as well as ongoing anxiety in patients after surgery [3]. The prevalence of PONV in surgical cases performed under general anesthesia may be between 30% and 80%. It is one of the most common and important problems that also reduce patient satisfaction [1]. In the literature, it is claimed that many demographic factors associated with PONV risk, as well as surgery type, anesthesia characteristics, anesthesia duration, postoperative opioid use, IV anesthetics, and inflammation, increase this risk [1,2].

Pneumothorax is characterized by shortness of breath and chest pain originating from the lungs. It may affect normal respiration due to the presence of air in the pleural space [4]. Pneumothoraxes are classified as spontaneous and non-spontaneous [5]. Primary spontaneous pneumothorax (PSP) typically occurs in young adults (highest incidence of age 20-30 years). Risk factors are male gender, smoking, and an asthenic body type. PSP usually presents with sudden chest pain or discomfort [5]. While PSP usually does not require immediate intervention, secondary spontaneous pneumothorax (SSP) is a potentially life-threatening circumstance due to decreased respiratory reserves because of the underlying lung disease [6]. 

It is a known fact that video-assisted thoracic surgery (VATS) offers significant clinical and economic advantages over open surgery in many clinical situations [7]. In addition, studies have shown that many procedures involving thoracotomy can be performed with VATS [7-10]. Although VATS is a less invasive surgical technique, postoperative pain may be a common condition. Failure to provide adequate postoperative analgesia causes postoperative atelectasis, limited inspiratory expansion of the thorax, and decreased patient comfort. This may also increase the need for additional analgesics and induce the emetic effect of these drugs [11]. PONV that may occur after VATS negatively affects patient comfort and may cause many problems such as dehydration, electrolyte imbalance, venous hypertension, esophageal rupture, separation of surgical suture lines, and life-threatening airway obstruction due to aspiration [12].

In the literature, many factors such as demographic characteristics of patients, duration of surgery, type of surgery, anesthetic and analgesic methods, and opioid use determine the incidence of PONV [13]. In addition, there are recent studies showing that inflammatory markers such as neutrophil-lymphocyte ratio (NLR) and platelet-lymphocyte ratio (PLR) can predict PONV [1,2].

We hypothesize that demographic and clinical data, as well as inflammatory markers such as NLR and PLR, may be effective in predicting PONV in patients undergoing VATS for pneumothorax.

In this study, we aimed to investigate the parameters that could predict PONV in patients who underwent VATS wedge resection for pneumothorax under standard anesthesia and analgesia method.

## Materials and methods

**Figure 1:**
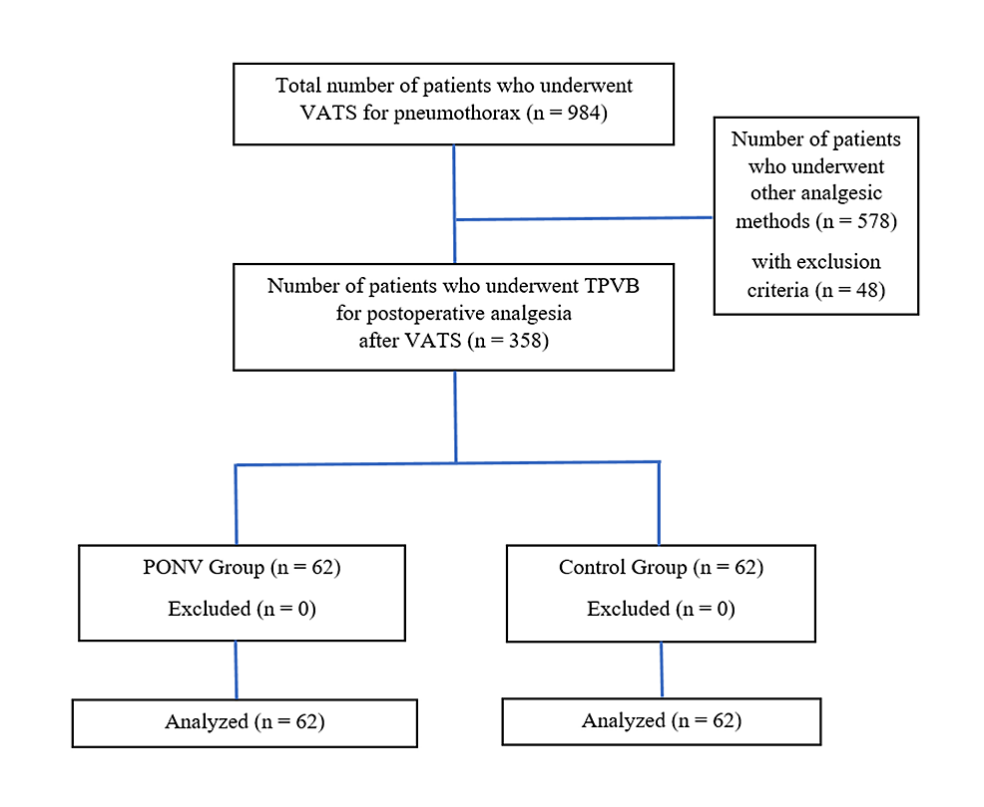
Flow charts of patients. VATS: Video-assisted thoracic surgery; TPVB: Thoracic Paravertebral Block; PONV: Postoperative nausea and vomiting.

Ankara Keçiören Training and Research Hospital Clinical Research Ethics Committee approval was obtained (ID: 2012-KEAK-15/2358, Date: 14.09.2021). Then, the data of patients who underwent elective VATS between January 1, 2018 and June 1, 2021 were analyzed retrospectively. Patients who underwent elective VATS/Wedge resection for pneumothorax, who were between the ages of 18-65, American Society of Anesthesiologists (ASA) I-III, and whose body mass indexes were between 18-30 kg/m2 were included in the study. According to medical records, patients under the age of 18 and over the age of 65, with ASA score of IV and above, with body mass index (BMI) below 18 kg/m2 and above 30 kg/m2, and patients who underwent surgery other than VATS/Wedge resection were excluded from the study. In addition, patients who received blood transfusion, used antiemetic and anticholinergic drugs, had continuous analgesics, and had a history of chronic pain were not included in the study. The patients were divided into two groups, the PONV group (Group 1) and the control group (Group 2) (Figure 1).

Patients' age, body mass index, gender, smoking history, ASA score, duration of anesthesia, preoperative and postoperative neutrophil, lymphocyte, platelet counts, NLR, PLR, postoperative first-hour and 24th-hour visual analog scale (VAS) scores, postoperative morphine and additional analgesic consumptions, and PONV incidence were recorded.

For general anesthesia induction, propofol (2 mg/kg), vecuronium (0.1 mg/kg), and fentanyl (1 µg/kg) were administered intravenously. Anesthesia was maintained by administering 2% sevoflurane in O2-air mixture and remifentanil infusion (0.01-0.20 µg/kg/min) in both groups. Additional IV doses vecuronium (of 0.03 mg/kg) were administered if needed. 10 mg IV metoclopramide was administered as an antiemetic and 100 mg IV tramadol with 50 mg IV dexketoprofen were administered for analgesia at the end of the surgery [14].

Thoracic paravertebral block (TPVB) procedure was performed under general anesthesia before the skin incision in order to prevent the patient's anxiety and ensure comfort. In the postoperative surgical intensive care unit, intravenous morphine was administered via a patient-controlled analgesia (PCA) pump for 24 hours. Pain intensity was evaluated using a 10-point VAS (0: No pain and 10: Unbearable pain). The PCA pump’s dose delivery was limited to administer a bolus dose of 1 mg of morphine and deliver a maximum dose of 12 mg of morphine in total within four hours with lockout intervals of 15 minutes [10]. For multimodal analgesia, all patients received paracetamol 1 g IV every eight hours, and 50 mg dexketoprofen IV was administered every 12 hours. Subsequently, 50 mg IV tramadol was given as an additional analgesic to patients with a VAS score of 4 and above [14].

Statistical analyses

The confounder effect was controlled using the Propensity Score Matching (PSM) analysis in order to distribute the case-control groups similarly in terms of statistical comparisons. Prior to the study, the confounder between the case-control groups was matched in terms of the continuous variables, age, gender, body mass index (BMI), ASA scores, smoking, and additional diseases [1]. Propensity scores were predicted using the combined estimator (ensemble learning: a combination of logistic regression and machine learning algorithms) developed by Demir et al. (2019) [15]. A matching analysis was performed using the Nearest Neighbor Matching method. The case-control ratio was matched equally as 1:1 in the matching analysis. After the matching analysis based on the propensity score, the control of the balance was evaluated using the overall chi-square balance test [16]. Before the matching, there were 296 patients in the control group and 62 patients in the PONV group. After the matching, analyses were performed with a total of 124 patients' data, with 62 patients distributed to two different groups equally according to confounder variables. A matching analysis was performed in the R package with the "Matching" library, and graphic drawings were done with the “ggplot 2” library [1,17].

Data analyses were performed by using Statistical Package for Social Sciences (SPSS) version 22.0 (IBM Corp., Armonk, NY, USA). Whether the distribution of continuous variables was normally distributed or not was determined by Kolmogorov Smirnov test. Levene test was used for the evaluation of homogeneity of variances. Unless specified otherwise, continuous data were described median (interquartile range) for skewed distributions. Categorical data were described as number of cases (%). Statistical analysis differences in not normally distributed variables between two independent groups were compared by Mann Whitney U test. Statistical analysis differences in not normally distributed variables between two dependent groups were compared by Wilcoxon test. Categorical variables were compared using Pearson’s chi-square test. A p-value of <0.05 was accepted as a significant level on all statistical analyses. Receiver operating characteristic (ROC) curve analysis was used to determine the cut-off value of the preoperative and postoperative NLR and preoperative and postoperative PLR associated with the risk of PONV.

## Results

The data of 984 patients who underwent VATS/Wedge resection for pneumothorax were analyzed retrospectively. Patients who used methods other than TPVB for postoperative analgesia (578 patients) and patients with exclusion criteria (48 patients) were excluded from the study (Figure 1).

There was no significant difference in terms of gender, age, BMI, smoking history, ASA score, and operation time between the groups (p > 0.05). Additional analgesic requirement and 24-hour morphine consumption were significantly higher in the PONV group (p: 0.005 and p < 0.001, respectively) (Table 1).

**Table 1:** Demographic data, additional analgesic use, duration of anesthesia, and 24-hour morphine consumption. Continuous variables were expressed as median (inter-quartile range) and categorical variables expressed as either frequency (percentage). Continuous variables were compared with Mann Whitney U test ^β^ and categorical variables were compared using Pearson’s chi-square test*. Statistically significant p-values were in bold. PONV: Postoperative nausea and vomiting; BMI: body mass index; ASA: American Society of Anesthesiologists.

		PONV Group (n:62)	Control Group (n:62)	p
Gender	Female	26 (41.9%)	21 (33.9%)	0.355 *
	Male	36 (58.1%)	41 (66.1%)	
Age, year		25.00 (11.00)	30.00 (14.00)	0.192^ β^
BMI kg/m^2^		21.80 (4.75)	22.93 (3.00)	0.169 ^β^
Smoking History		37 (59.7%)	34 (54.8%)	0.586 *
ASA	ASA I	15 (24.2%)	16 (25.8%)	0.972 *
	ASA II	21 (33.9%)	20 (32.3%)	
	ASA III	26 (41.9%)	26 (41.9%)	
Additional Analgesic Use		40 (64.5%)	24 (38.7%)	0.004 *
Duration of Anesthesia (minute)		120.00 (0)	120.00 (0)	0.124^ β^
Morphine Consumption (mg)		27.00 (18)	13.00 (17)	< 0.001^ β^

In Table 2, the PONV group and the control group are compared between each other and within the group according to the preoperative and postoperative hemogram results.

**Table 2:** Comparison of hemogram parameters and VAS scores between and within the groups. Continuous variables were expressed as mean ± SD and median (interquartile range). Continuous independent variables were compared with Mann Whitney U test. Continuous dependent variables were compared with Wilcoxon test. Statistically significant p-values were in bold. PONV: Postoperative nausea and vomiting; RDW: Red cell distribution; NLR: neutrophil-lymphocyte ratio; PLR: platelet-lymphocyte ratio; VAS: visual analog scale; IQR: interquartile range; SD: standard deviation.

		PONV Group (n:62)		Control Group (n:62)		P value (Independent)
Neutrophil (x10^ᴧ^3/µL)						
Preoperative		8.27	(3.82)	4.65	(1.69)	<0.001
Postoperative		12.20	(3.64)	7.90	(2.65)	<0.001
P value (dependent)		<0.001		<0.001		
Lymphocyte (x10^ᴧ^3/µL)						
Preoperative		1.80	(0.66)	2.09	(0.66)	0.020
Postoperative		1.16	(0.71)	1.46	(0.70)	0.013
P value (dependent)		<0.001		<0.001		
Platelet (x10^ᴧ^3/µL)						
Preoperative		273.00	(70.00)	265.00	(119.00)	0.667
Postoperative		227.00	(124.00)	228.00	(88.00)	0.956
P value (dependent)		<0.001		<0.001		
RDW (%)						
Preoperative		13.60	(1.10)	13.40	(1.80)	0.966
Postoperative		13.40	(1.20)	13.35	(2.00)	0.755
P value (dependent)		0.002		0.008		
NLR						
Preoperative		4.48	(3.13)	2.14	(1.44)	<0.001
Postoperative		9.83	(5.22)	5.99	(4.66)	<0.001
P value (dependent)		<0.001		<0.001		
PLR						
Preoperative		148.89	(85.57)	117.14	(64.16)	0.022
Postoperative		187.50	(128.9)	154.12	(87.72)	0.081
P value (dependent)		<0.001		<0.001		
VAS at rest						
1^st^ hour	Median (IQR)	4.00	(2.00)	3.00	(2.00)	0.004
	Mean ± SD	4.02	±1.38	3.18	±1.65	
24^th^ hour	Median (IQR)	2.00	(1.00)	2.00	(2.00)	<0.001
	Mean ± SD	2.46	±1.16	1.56	±1.26	
P value (dependent)		<0.001		<0.001		

Preoperative neutrophils (p < 0.001), postoperative neutrophils (p < 0.001), preoperative NLR (p < 0.001), postoperative NLR (p < 0.001), preoperative PLR (p: 0.022) were statistically significantly higher in the PONV group compared to the control group (Table 2).

Preoperative and postoperative lymphocytes were statistically significantly lower in the PONV group compared to the control group (p: 0.020 and p: 0.013, respectively) (Table 2).

**Figure 2:**
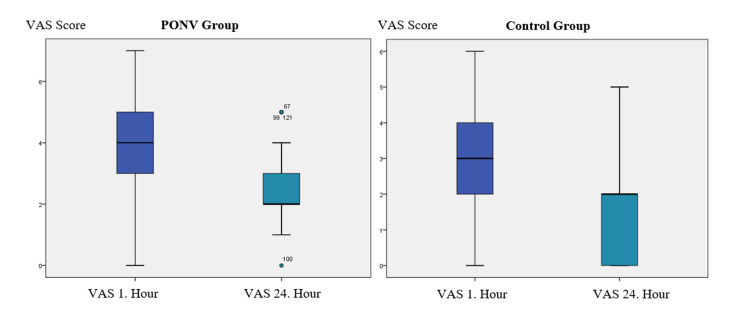
Box plot graphs of the VAS scores at postoperative 1st hour and 24th hour. PONV: Postoperative nausea and vomiting; VAS: Visual analog scale.

VAS scores were statistically significantly higher in the PONV group at the first hour and at the 24th hour compared to the control group (p: 0.004 and p <0.001, respectively) (Table 2) (Figure 2).

**Figure 3:**
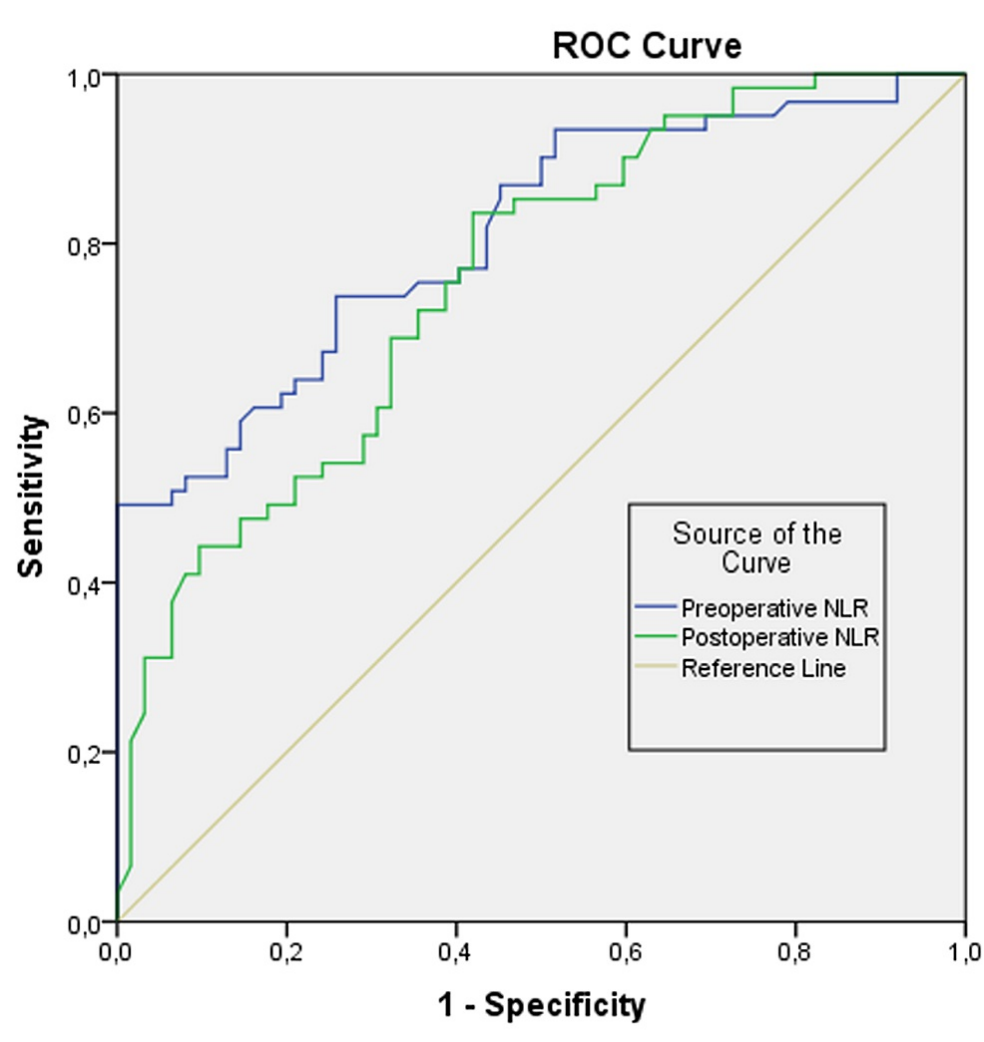
ROC curves obtained for the predicting success of preoperative and postoperative NLR parameter in a PONV diagnosis. ROC: receiver operating characteristic; NLR: neutrophil-lymphocyte ratio; PONV: postoperative nausea and vomiting.

In the ROC analysis, the area under the procedure characteristic curve (AUC) for preoperative and postoperative NLR was calculated as 0.811 and 0.757, respectively, and was statistically significant (p < 0.05) (Figure 3). This result shows that preoperative and postoperative NLR level can distinguish between patients with and without nausea. In order to answer the question of which value should be taken as the cut-off value for the preoperative and postoperative NLR groups, the sensitivity and specificity values given as a result of the analysis were examined and optimum points were selected. When the cut-off value was accepted as 3.01 in terms of preoperative NLR level, the sensitivity was calculated as 73.8%, the specificity as 74.2%. PONV rate is higher in cases with preoperative NLR > 3.01. When the cut-off value was accepted as 7.13 in terms of postoperative NLR level, the sensitivity was calculated as 83.6%, the specificity as 58.1%. PONV rate is higher in cases with postoperative NLR > 7.13 (Table 3).

**Table 3:** ROC curve results and sensitivity, specificity, positive-negative predictive, and likelihood ratio (+) values. ROC: receiver operating characteristic; NLR: neutrophil-to-lymphocyte ratio; AUC: area under curve, PPV: positive predictive value, NPV: negative predictive value, LR+: positive likelihood ratio, LR-: negative likelihood ratio

	Preoperative NLR	Postoperative NLR
AUC (95% CI)	0.811 (0.736-0.886)	0.757 (0.674-0.841)
P values	<0.001	<0.001
Cut Off	3.01	7.13
Sensitivity	%73.8	%83.6
Specificity	%74.2	%58.1
PPV	%73.8	%66.2
NPV	%74.2	%78.3
LR+	2.86	1.99
LR-	0.35	0.28

**Figure 4:**
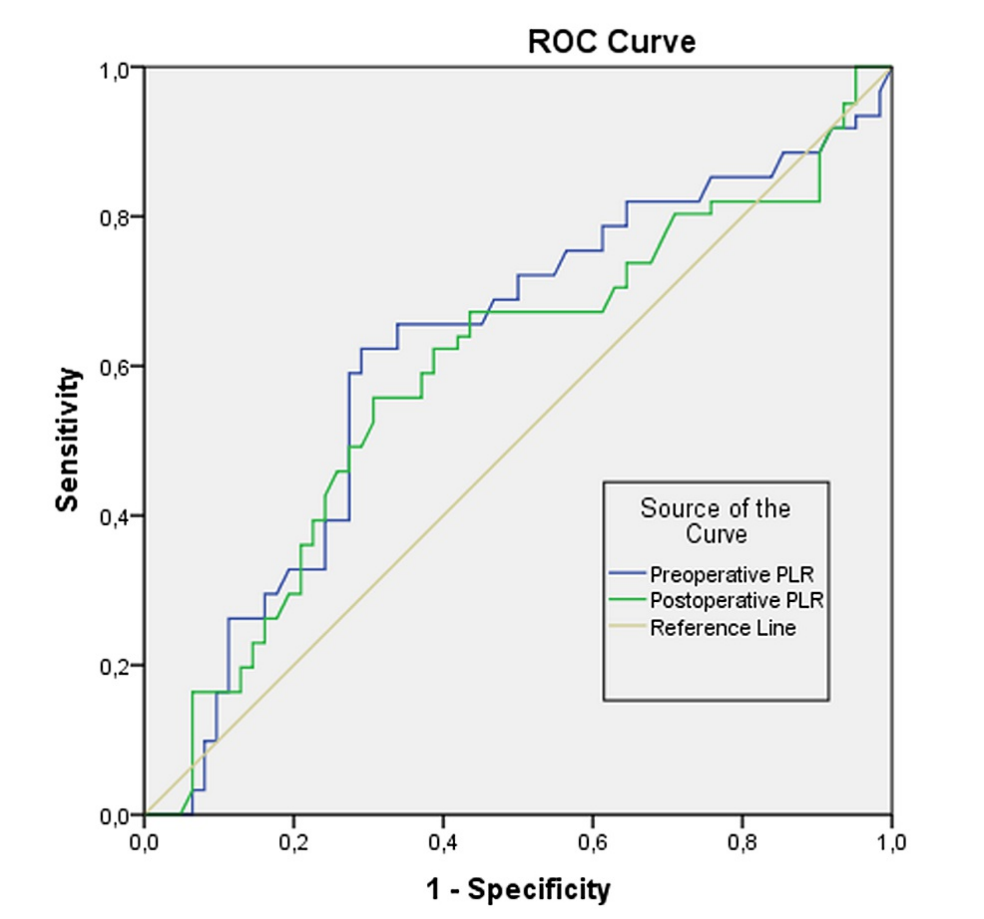
ROC curves obtained for the predicting success of preoperative and postoperative PLR parameter in a PONV diagnosis. ROC: receiver operating characteristic; PLR: platelet-lymphocyte ratio; PONV: postoperative nausea and vomiting.

In the ROC analysis, the area under the operation characteristic curve (AUC) for preoperative and postoperative PLR was calculated as 0.620 and 0.591, respectively, and the preoperative PLR was found to be statistically significant (p<0.05) (Figure 4). This result shows that the preoperative PLR level can distinguish between patients with and without nausea. In order to answer the question of which value should be taken as the cut-off value for preoperative PLR groups, the sensitivity and specificity values given as a result of the analysis were examined and optimum points were selected. When the cut-off value was accepted as 140.39 in terms of preoperative PLR level, the sensitivity was calculated as 62.3%, specificity as 71.0%. In cases with preoperative PLR > 140.39, the rate of cases with vomiting is higher (Table 4).

**Table 4:** ROC curve results and sensitivity, specificity, positive-negative predictive, and likelihood ratio (+) values. ROC: receiver operating characteristic; PLR: Platelet-to-lymphocyte ratio; AUC: area under curve, PPV: positive predictive value, NPV: negative predictive value, LR+: positive likelihood ratio, LR-: negative likelihood ratio

	Preoperative PLR	Postoperative PLR
AUC (95% CI)	0.620 (0.518-0.721)	0.591 (0.489-0.693)
P values	0.022	0.081
Cut Off	140.39	-
Sensitivity	% 62.3	-
Specificity	% 71.0	-
PPV	% 67.9	-
NPV	% 65.7	-
LR+	2.15	-
LR-	0.53	-

## Discussion

The results of this study showed that neutrophil and NLR were significantly higher in patients with PONV, and VAS scores, and accordingly, postoperative analgesic needs were found to be higher in these patients. In addition, the preoperative PLR, preoperative and postoperative NLR values above 140.39, 3.01 and 7.13 may be a guide in predicting PONV, respectively. This study is particular in terms of evaluating PONV in a specific group of surgery with similar demographic data and postoperative analgesia management.

PONV is an undesirable condition that is still up to date as a very serious and unpleasant postoperative side effect for patients. This situation is related to many factors, including surgery, anesthesia management, and medical treatments, as well as patient characteristics [1,2,18]. The prevalence of PONV in surgical cases performed under general anesthesia may be between 30% and 80%. It is one of the most common and important problems that also reduce patient satisfaction [1]. The complex physiology of PONV causes the mechanism to be not fully understood. According to the modeling of Pierre et al. [18], the brain structures involved in the pathophysiology of vomiting are not centralized in an anatomically defined 'vomiting center' and are distributed throughout the medulla oblongata. The chemoreceptor trigger zone (CRTZ) receives input from vagal afferents in the gastrointestinal tract, and emetogenic toxins, metabolites, and drugs circulating in the blood and cerebrospinal fluid act by crossing the blood-brain barrier [18]. Neurons in the CRTZ project to the nucleus tractus solitarius, which receives input from the vagal afferents and the vestibular and limbic systems, which triggers vomiting by stimulating the ventral respiratory group and the dorsal motor nucleus of the vagus [18].

Different scoring systems are used to predict PONV, and they are mainly based on patients' demographic data and clinical history [12,13,19]. In a study by Moreno et al. [20], they stated that previous history of PONV and female gender were independent risk factors in predicting postoperative nausea and vomiting. In our study, we aimed to find the clinical and laboratory parameters affecting PONV by determining demographic data on similar patients using the propensity score matching method. In the risk scoring of Apfel et al. [13], female gender, PONV/motion sickness history, non-smoking status, and postoperative opioid use are evaluated. Studies investigating the relationship of inflammatory parameters such as NLR and PLR with PONV are limited [1,2]. In this study, the effects of inflammatory parameters in a specific surgical group were investigated and the relationship between NLR and PONV was found to be significant. This supports the argument that in addition to demographic data, inflammatory parameters measured in the preoperative and postoperative period may also be effective in predicting PONV.

While the cut-off values determined for NLR in predicting PONV in previous studies were generally around 2 [1,2,21], this value was found to be 3.01 in the preoperative period and 7.13 in the postoperative period in our study. This difference may be related to the stress response of the patients and the inflammatory response to chest tube application in pneumothorax cases. This suggests that a disease-based evaluation may be appropriate when determining the cut-off value for NLR to predict PONV.

In a study by Karaca et al. [1], they showed that PLR could be an indicator for predicting PONV as well as NLR. While there are studies investigating the relationship between PONV and NLR, studies investigating the effect of PLR on PONV are very limited [1]. In this study, while PLR values were significantly different in the preoperative period, similar results were found in the postoperative period in groups. In conclusion, the effect of PLR in predicting PONV was limited in our study.

Another important result of this study is the significantly higher VAS scores and opioid consumption in the patients who developed PONV. It is a known fact that additional analgesia or opioid consumption increases when patients cannot provide adequate pain control. This condition can also cause an uncontrolled stress response. In addition, increased consumption of opioids to control pain may cause PONV [19,22]. Although VAS scores were tried to be kept below 4 with multimodal analgesia particularly first hours of the postoperative period, the amount of additional analgesic applied to ensure this situation was higher in patients who developed PONV. This situation shows that the stress response is high and the inflammatory process is more pronounced in this patient group, as well as explaining the high level of PONV due to the emetogenic effect of these drugs due to the use of additional analgesics.

We have some limitations in this study. First of all, the study is retrospective and single-center. In this study, a similar distribution of confounding factors, which reduced bias in retrospective studies, was achieved by using propensity score matching. In addition, since the chest tube duration and pain/anxiety status of the patients in the preoperative period could not be analyzed in the study, the effect of this situation on inflammatory parameters could not be fully evaluated.

## Conclusions

In conclusion, NLR derived from simple and routine hemogram parameters can be an effective parameter with high sensitivity and specificity in predicting PONV, which has a negative impact on patients' outcome in the postoperative period. Besides, preoperative PLR may also be effective in predicting PONV. A treatment that can be planned according to these parameters can play a key role in preventing PONV. In addition, efficient perioperative analgesia management may be effective in reducing PONV by limiting the emetogenic analgesics used.
